# Kindlin-3 in platelets and myeloid cells differentially regulates deep vein thrombosis in mice

**DOI:** 10.18632/aging.102229

**Published:** 2019-08-31

**Authors:** Yanyan Yan, Hongqin Yang, Xiao Hu, Zeping Zhang, Shushu Ge, Zhen Xu, Juan Gao, Junling Liu, Gilbert C. White, Yan-Qing Ma

**Affiliations:** 1 Collaborative Research Program for Cell Adhesion Molecules, Shanghai University School of Life Sciences, Shanghai, China; 2 Blood Research Institute, Versiti, Milwaukee, WI 53213, USA; 3 Department of Biochemistry and Molecular Cell Biology, Shanghai Jiao-Tong University School of Medicine, Shanghai, China; 4 Department of Biochemistry, Medical College of Milwaukee, Milwaukee, WI 53226, USA

**Keywords:** kindlin-3, platelets, neutrophils, deep vein thrombosis, NETs

## Abstract

Platelets and myeloid cells cooperate to promote deep vein thrombosis (DVT). Here we evaluated the role of kindlin-3, a key integrin activator in these cells, in regulating stenosis-induced DVT in mice. DVT was significantly suppressed in mice that express a kindlin-3 mutant defective for integrin binding, showing that kindlin-3-mediated integrin signaling in blood cells is required for DVT. While platelet-specific deficiency of kindlin-3 in Kindlin-3^fl/fl^PF4-Cre mice significantly suppressed DVT, deficiency of kindlin-3 specifically in myeloid cells in Kindlin-3^fl/fl^LysM-Cre mice remarkably enhanced the early development of DVT, indicating that kindlin-3 in platelets and myeloid cells can play distinct roles in regulating DVT. Mechanistically, the levels of neutrophil extracellular traps (NETs) in plasma, a key DVT facilitator, were significantly elevated in Kindlin-3^fl/fl^LysM-Cre mice upon the IVC stenosis; and treatment with either DNase I or PAD4 inhibitor could effectively compromise the enhancement of DVT in these mice, suggesting that kindlin-3 in neutrophils may affect DVT via restraining NET release. In addition, we found that the kindlin-3-integrin αIIbβ3 signaling in platelets was required to promote NET release. Together, our studies reveal that kindlin-3 in platelets and myeloid cells can differentially regulate DVT through orchestrating NET release, thus providing further mechanistic insights into DVT.

## INTRODUCTION

Deep vein thrombosis (DVT) and associated pulmonary embolism are life-threatening complications. About 1 in 1000 adults develops DVT annually and aging increases its rate of occurrence. Recent studies in mice show that platelets and myeloid cells (neutrophils and monocytes) cooperate to initiate and propagate DVT [1]. DVT is pathologically associated with many inflammatory conditions, such as acute infections [2], autoimmune disorders [3], and late-stage cancers [4]. Neutrophils are the most abundant and the first leukocytes to be recruited to the sites of infections and inflammation, and they are able to release neutrophil extracellular traps (NETs) as one of the key pathogen-killing mechanisms [5]. NETs are scaffolds of chromatin fibers released from inflamed neutrophils that are decorated with both antimicrobial and pro-coagulation proteins [1, 5, 6], thus being able to mediate the crosstalk between the innate immune response and thrombosis. In fact, the levels of NETs in plasma are correlated with DVT in both animal models and human patients [7–10]. In addition, many pathological conditions associated with high-risk of DVT are often concomitant with elevated plasma NETs [11]. Treatment with DNase I can effectively suppress DVT in mouse models [1, 7]. Therefore, NETs contribute to DVT. Particularly, it has been reported that activated platelets also play a role in supporting NET release [12–14].

Kindlin-3 is predominantly expressed in platelets and leukocytes [15–18]. Deficiency of kindlin-3 in humans results in type III leukocyte adhesion deficiency (LAD-III), featured with defects of both platelet aggregation and leukocyte adhesion. Mechanistically, kindlin-3 in these cells interacts with the integrin β cytoplasmic tails (CTs) to support integrin activation and integrin-mediated cell adhesion [18–20]. Interaction-disrupting mutations in either kindlin-3 or the integrin β CTs lead to LAD-III like phenotypes in mice [21–23]. Kindlin can also perform integrin-independent functions [24, 25]. For example, our previous study has demonstrated that kindlin-3 in neutrophils can negatively regulate ROS/NET release, which seems to be independent of its binding to integrin [25]. Collectively, kindlin-3 can play multifaceted roles in different blood cells.

In this study, we evaluate the respective and reciprocal roles of kindlin-3 in platelets and myeloid cells in DVT, and reveal that kindlin-3 in these cells can differentially regulate DVT in mice through orchestrating NET release, providing novel insights into the mechanistic details of DVT.

## RESULTS

### Kindlin-3 in platelets and myeloid cells plays distinct roles in regulating stenosis-induced DVT in mice

**Figure 1:**
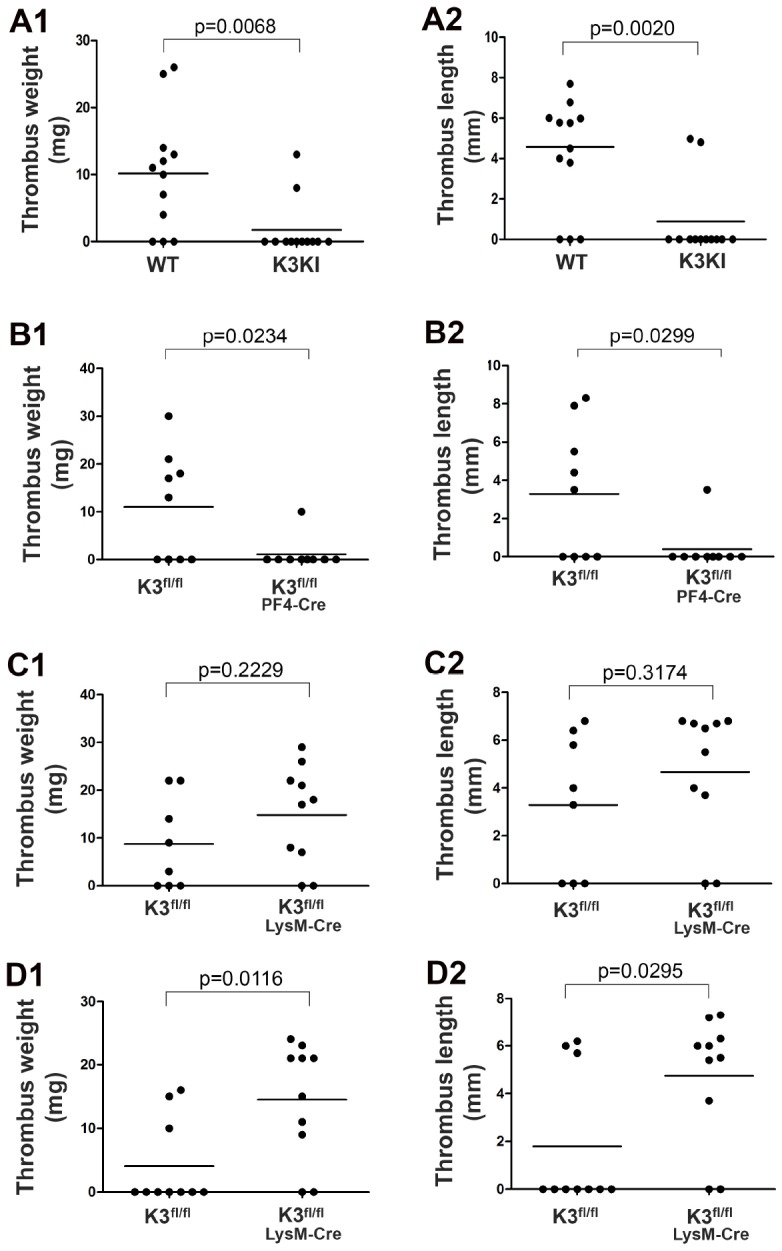
**Determine the role of kindlin-3 in platelets and myeloid cells in regulating stenosis-induced DVT in mice.** (**A1**–**A2**) K3KI mice and wild type littermates were subjected to partial IVC ligation for 48 hours. After that, the IVC tissues were harvested, and thrombus weight and length were evaluated; n = 12 for each group. (**B1**–**B2**) Thrombus formation was evaluated in Kindlin-3^fl/fl^PF4 mice and Kindlin-3^fl/fl^ littermates by partially ligating the IVC for 48 hours; n = 9 for each group. (**C** and **D**) Partial IVC ligation was applied to Kindlin-3^fl/fl^LysM-Cre mice and Kindlin-3^fl/fl^ littermates for either 48 hours (**C1**–**C2**) or 6 hours (**D1**–**D2**), and thrombus formation in these mice was evaluated; n ≥ 8 for each group. Dots represent individual experiments for each mouse and lines in dot plots represent mean. A value of P < 0.05 was considered significant.

Functional involvement of kindlin-3 in DVT was first evaluated in kindlin-3 knock-in mice (K3KI) mice that express a kindlin-3 mutant defective for integrin binding. Here we utilized a widely used stenosis-induced DVT model in mice by partially ligating the IVC [26]. As shown in Figure 1A1-A2, thrombi were dominantly formed in the IVC in in-bred wild type mice after partial ligation for 2 days while most of K3KI mice failed to develop thrombi under the same condition, suggesting that disconnection between kindlin-3 and integrin in blood cells can significantly compromise stenosis-induced DVT. Interestingly, once thrombi were formed in K3KI mice, the magnitudes of thrombus weights and lengths were actually comparable to wild type counterparts, indicating that the kindlin-3-integrin signaling in blood cells may preferentially advance the early stage of DVT in mice.

Both platelets and myeloid cells are able to be recruited to the IVC wall upon stenosis [1]. We next sought to evaluate the respective role of kindlin-3 in these cells in DVT. First, we bred Kindlin-3^fl/fl^ mice with PF4-Cre mice to obtain Kindlin-3^fl/fl^PF4-Cre mice with specific deficiency of kindlin-3 in platelets (Supplementary Figure 1). As shown in Figure 1B1-B2, Kindlin-3^fl/fl^PF4-Cre mice exhibited significantly compromised DVT when compared to control Kindlin-3^fl/fl^ mice after partial ligation of the IVC for 2 days, showing that kindlin-3 in platelets plays an essential role in promoting DVT. In addition, transfusion of Kindlin-3^fl/fl^PF4-Cre mice with platelets isolated from wild type mice could restore DVT (data not shown), demonstrating that the kindlin-3-integrin signaling in platelets is required to support the development of DVT in mice.

Interestingly, after partially ligating the IVC for 2 days, Kindlin-3^fl/fl^LysM-Cre mice with a specific deficiency of kindlin-3 in myeloid cells significantly developed DVT (Figure 1C1-C2); when compared to in-bred Kindlin-3^fl/fl^ mice, DVT in Kindlin-3^fl/fl^LysM-Cre mice actually exhibited a tendency of enhancement although there was no statistical significance. When the condition of stenosis was reduced to 6 hours, the enhanced DVT in Kindlin-3^fl/fl^LysM-Cre mice became significant (Figure 1D1-D2). Even at a 2-hour time point of stenosis, under which thrombi were scarcely formed in control Kindlin-3^fl/fl^ mice, Kindlin-3^fl/fl^LysM-Cre mice still could significantly develop DVT (Supplementary Figure 2). Together, these results indicate that deficiency of kindlin-3 in myeloid cells may facilitate the early stage of development of DVT in mice.

Collectively, the above findings disclose that kindlin-3 in platelets and myeloid cells can play distinct roles in regulating the development of DVT in mice, revealing a novel and complex regulation of the kindlin-3 signaling in venous thrombosis.

### Kindlin-3 in platelets and neutrophils differentially orchestrates NET release in DVT

**Figure 2:**
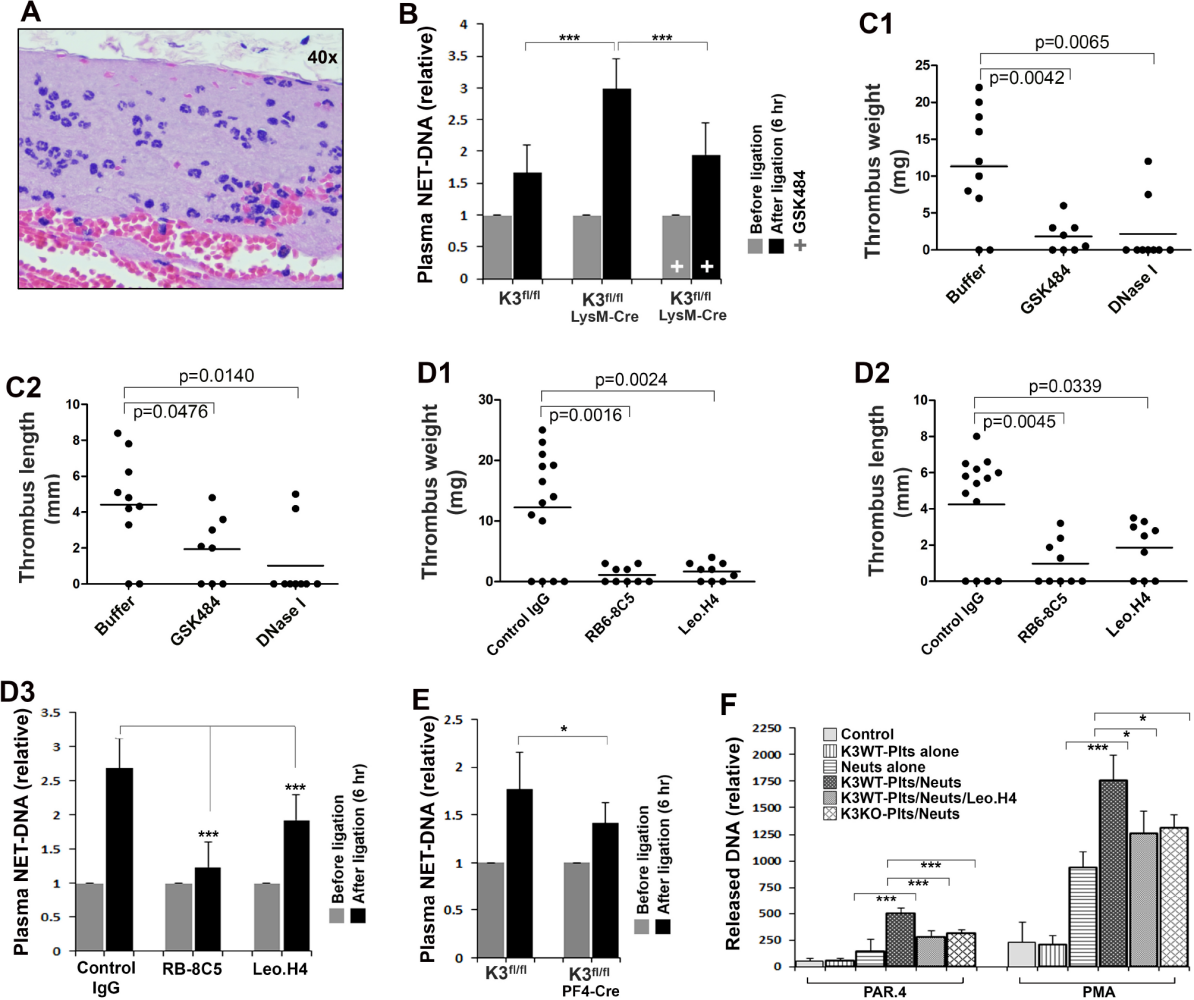
**Determine the role of kindlin-3 in platelets and neutrophils in regulating NET release *in vivo* and *in vitro*.** (**A**) Histological analysis of the thrombi formed in the IVC in Kindlin-3^fl/fl^LysM-Cre mice after partial ligation for 6 hours. (**B**) The levels of NETs in plasma were measured using an ELISA-based assay as described in methods. Data are shown as mean ± SD; n ≥ 8 for each group. (**C1**–**C2**) Kindlin-3^fl/fl^LysM-Cre mice were injected with DNase I, GSK484 or buffer, and the partial IVC ligation was performed. Thrombus formation in the IVC was determined 6 hours after the ligation. Dots represent individual experiments for each mouse and lines in dot plots represent mean; n ≥ 8 for each group. (**D1**–**D3**) After treatment with the indicated antibodies, Kindlin-3^fl/fl^LysM-Cre mice were subjected to partial IVC ligation for 6 hours and then the IVC tissues were harvested and evaluated (**D1**–**D2**). Dots represent individual experiments for each mouse and lines in dot plots represent mean; n ≥ 8 for each group. Meanwhile, blood samples were collected from mice before and after the IVC stenosis and used for measuring plasma NETs by the ELISA-based assay. Data are shown as mean ± SD. (**E**) blood samples were collected from Kindlin-3^fl/fl^PF4-Cre mice and Kindlin-3^fl/fl^ mice before and after the IVC stenosis and used for measuring plasma NETs by the ELISA-based assay (**D3**). Data are shown as mean ± SD. (**F**) Mouse bone marrow neutrophils (Neuts) were incubated with TNF-α primed endothelial cell monolayers with or without the presence of washed platelets isolated from either Kindlin-3^fl/fl^ mice (K3WT-Plts) or Kindlin-3^fl/fl^PF4 mice (K3KO-Plts). After stimulation with either PAR4 agonist peptide (150 μM) or PMA (20 nM) for 2 hours in the presence or absence of Leo.H4 antibody, the released NETs were quantified as described in methods. Data are shown as mean ± SD. (*, *p<0.05*; ***, *p<0.001*).

The enhanced DVT in Kindlin-3^fl/fl^LysM-Cre mice at the early stage indicates that kindlin-3 in myeloid cells is actually able to limit the development of DVT. To further clarify the participation of myeloid cells in DVT, we histologically analyzed the thrombi collected from Kindlin-3^fl/fl^LysM-Cre mice. Interestingly, we found that, in the 2-day stenosis model, the recruited myeloid cells in thrombi formed in Kindlin-3^fl/fl^LysM-Cre mice were relatively comparable to those in control Kindlin-3^fl/fl^ mice (data not shown). Further, we found that in thrombi formed at the early stage of stenosis in Kindlin-3^fl/fl^LysM-Cre mice, a condition under which thrombi were dominantly formed only in Kindlin-3^fl/fl^LysM-Cre mice but not in control Kindlin-3^fl/fl^ mice, accumulated myeloid cells were prevalent and these cells were exclusively neutrophils (Figure 2A). These findings suggest that neutrophils with deficiency of kindlin-3 may alternatively employ an integrin-independent mechanism to mediate neutrophil recruitment into the thrombi formed in Kindlin-3^fl/fl^LysM-Cre mice.

Previously, we found that presence of kindlin-3 in neutrophils is able to restrict NET release [25]. Since NETs act as a potent DVT promoter in mice, we therefore sought to investigate how kindlin-3 in neutrophils orchestrates NET release in Kindlin-3^fl/fl^LysM-Cre mice in the early stenosis model. As shown in Figure 2B, after partially ligating the IVC for 6 hours, the levels of plasma NETs in Kindlin-3^fl/fl^LysM-Cre mice were significantly higher than those in control Kindlin-3^fl/fl^ mice and the elevation of plasma NETs in Kindlin-3^fl/fl^LysM-Cre mice could be inhibited by treatment with GSK484, an inhibitor of NET formation, disclosing that deficiency of kindlin-3 in neutrophils can promote NET release *in vivo*. Substantially, treatment with either DNase I to digest NETs or GSK484 (a PAD4 inhibitor) to inhibit NET release in Kindlin-3^fl/fl^LysM-Cre mice greatly suppressed DVT (Figure 2C1 and C2), demonstrating that the enhanced DVT in Kindlin-3^fl/fl^LysM-Cre mice at the early stage is actually facilitated by NETs. In addition, the role of neutrophils in enhancing NET release and DVT in Kindlin-3^fl/fl^LysM-Cre mice was verified by neutrophil depletion (Figure 2, D1–D3). Collectively, these results imply that the elevated levels of NETs in Kindlin-3^fl/fl^LysM-Cre mice upon stenosis may play a key role in facilitating the early development of DVT.

Importantly, DVT in Kindlin-3^fl/fl^LysM-Cre mice in the early stenosis model was significantly suppressed by platelet depletion that was realized by treating mice with Leo.H4, an antibody for integrin αIIbβ3 (Figure2D1 and D2). The suppressed DVT was concomitant with decreased NET release (Figure 2, D3). In addition, functional blockage of integrin αIIbβ3 without platelet depletion in Kindlin-3^fl/fl^LysM-Cre mice by the F(ab’)2 fragment of Leo.H4 antibody could also suppress DVT (data not shown). Combined with the previous results as shown in Figure 1B1 and B2, these findings suggest that the kindlin-3-integrin αIIbβ3 signaling in platelets is essentially required to facilitate NET release and DVT in mice.

To further verify the role of kindlin-3 in platelets in supporting NET release, we quantified plasma NETs in Kindlin-3^fl/fl^PF4-Cre mice and control Kindlin-3^fl/fl^ mice upon the IVC stenosis. As shown in Figure 2E, after partially ligating the IVC for 6 hours, the levels of plasma NETs in Kindlin-3^fl/fl^PF4-Cre mice significantly decreased when compared to Kindlin-3^fl/fl^ mice, indicating that kindlin-3 in platelets is involved in promoting NET release *in vivo*. Moreover, an *in vitro* NET release experiment was performed, in which bone marrow neutrophils were isolated and incubated with inflamed endothelial monolayer in presence of platelets isolated from either wild type or kindlin-3-deficient mice. As shown in Figure 2F, presence of platelets isolated from Kindlin-3^fl/fl^ mice significantly enhanced NET release upon stimulation with either PAR4 agonist peptide or PMA, and the enhanced NET formation could be significantly inhibited by a blocking antibody for integrin αIIbβ3. In addition, stimulation with collagen exhibited a similar pattern with PAR4, whereas a negligible effect was detected upon stimulation with ADP alone (data not shown), suggesting that strong platelet activation may be required to execute the promotion on NET release. Significantly, when compared to wild type platelets, kindlin-3-deficient platelets isolated from Kindlin-3^fl/fl^PF4-Cre mice exhibited a compromised ability to promote NET release under the same condition. Hence, these results verify that the kindlin-3-integrin αIIbβ3 signaling in activated platelets is required to support NET formation.

Taken all together, these findings reveal that kindlin-3 in platelets and neutrophils can differentially influence the outcome of DVT by orchestrating NET release.

## DISCUSSION

DVT and the associated complications are frequent causes of morbidity and mortality. Even though the causative factors of DVT have been described extensively in the literature, the detailed molecular mechanisms regulating the development of DVT still remain largely undisclosed. Recent studies show that local recruitment of platelets and myeloid cells promotes DVT in mouse models, in which NET release has an important impact [1, 7, 27]. Kindlin-3, a key integrin activator, is expressed in both platelets and myeloid cells. Therefore in this study we sought to explore the regulatory role of kindlin-3 in platelets and myeloid cells in stenosis-induced DVT in mice and the underlying mechanistic details. The functional involvement of the kindlin-3-integrin signaling in hematopoietic cells in DVT is clearly evidenced in K3KI mice that are resistant to DVT (Figure 1A). Interestingly, kindlin-3 in platelets and myeloid cells can differently influence the outcome of DVT. Deficiency of kindlin-3 in platelets significantly impairs DVT (Figure 1B), which is likely through crosstalk with integrin αIIbβ3 (Figure 2F). However, deficiency of kindlin-3 in myeloid cells unexpectedly promotes the early development of DVT (Figure 1D). Our previous work has demonstrated that deficiency of kindlin-3 in neutrophils can possess both pro- and anti-DVT properties, by promoting NET release and by inhibiting neutrophil recruitment, respectively [22, 25]. As a matter of fact, the significant enhancement of the early-stage DVT in Kindlin-3^fl/fl^LysM-Cre mice, as observed in this study, suggests that the pro-DVT feature is dominant in these mice. Although kindlin-3 is required to support integrin-mediated neutrophil adhesion and migration, we unexpectedly observe that neutrophils are still prevalently present in the early formed IVC thrombi in Kindlin-3^fl/fl^LysM-Cre mice (Figure 2A). This finding may imply that kindlin-3-deficient neutrophils possibly utilize a compensatory mechanism to facilitate neutrophil recruitment into thrombi in Kindlin-3^fl/fl^LysM-Cre mice, but the mechanistic details need to be further investigated.

Further, we demonstrate that the enhanced DVT at the early stenosis stage in Kindlin-3^fl/fl^LysM-Cre mice is contributed by the increased NET release in these mice. First, the levels of plasma NETs in Kindlin-3^fl/fl^LysM-Cre mice are significantly elevated after partial ligation of the IVC (Figure 2B). Second, inhibition of NET release by treatment with GSK484 (a PAD4 inhibitor) or digestion of NETs with DNase I in Kindlin-3^fl/fl^LysM-Cre mice both significantly inhibits DVT (Figure 2C). Third, neutrophil depletion in Kindlin-3^fl/fl^LysM-Cre mice suppresses DVT (Figure 2C). In addition, the kindlin-3-αIIbβ3 signaling in platelets plays an important role in promoting both DVT and NET release (Figure 2D) [1, 28, 29]. Nonetheless, the involvement of NETs may not be the sole mechanism in supporting DVT in Kindlin-3^fl/fl^LysM-Cre mice due to the fact that deficiency of kindlin-3 can have multiple consequences in neutrophils [25, 30]. Additional studies, especially to identify the key signaling factors downstream of kindlin-3 in neutrophils, are necessary for further interpreting the mechanistic details.

In summary, we demonstrate that the kindlin-3 mediated integrin αIIbβ3 signaling in platelets is prerequisite for facilitating the development of DVT. In addition, we also prove that deficiency of kindlin-3 in myeloid cells significantly advances the early development of DVT. Mechanistically, the regulatory role of kindlin-3 in platelets and myeloid cells in DVT are through affecting NET release. Collectively, our findings in this study further delineate the dynamic role of the interface between thrombosis and inflammation in influencing DVT, which may benefit the future development of comprehensive ant-DVT strategies.

## MATERIALS AND METHODS

### Kindlin-3 mice

Kindlin-3 knock-in (K3KI) mice and floxed kindlin-3 mice (Kindlin-3^fl/fl^) were generated as previously described [21, 25]. K3KI mice express a kindlin-3 mutant that is defective for integrin binding. Kindlin-3^fl/fl^ mice were used to breed with PF4-Cre mice and LysM-Cre mice to obtain Kindlin-3^fl/fl^PF4-Cre mice and Kindlin-3^fl/fl^LysM-Cre mice, with specific deficiency of kindlin-3 in platelets and myeloid cells, respectively. All animal studies were approved by the IACUC.

### Stenosis-induced DVT model

Mice within 8–12 weeks were anesthetized by isoflurane-oxygen inhalation. A laparotomy was performed to expose the inferior vena cava (IVC). The IVC was carefully separated from the attached tissues at the area just below the renal veins and ligated over a spacer (5.0 monofilament polypropylene filament). After ligation, the spacer was carefully removed to avoid complete vessel occlusion. Meanwhile, back branches were either ligated or cauterized. Peritoneum and skin were immediately closed and sutured. At the defined time points, mice were sacrificed and the IVC tissues were collected for further quantification.

### NET inhibition, platelet and neutrophil depletion and platelet αIIbβ3 blockage *in vivo*

DNase I (Fermentas) and GSK484 (MCE), a PAD4 inhibitor, were used to reduce NETs *in vivo*. DNase I was intravenously injected into mice approximately 30 min before the IVC ligation procedure at a dose of 120 U per mouse. GSK484 was intraperitoneally administrated twice with a 20-hour interval before the IVC ligation at dose of 4 mg/kg. Sterile vehicle buffer was used to inject mice as controls. Neutrophil depletion and platelet depletion in mice were realized by intravenous injection of an anti-Ly6G antibody RB6-8C5 (Bio X Cell) at a dose of 250 μg per mouse and an anti-αIIbβ3 antibody Leo.H4 (Emfret) at a dose of 20 μg per mouse, respectively. Functional blockage of integrin αIIbβ3 in mice was realized by intravenous injection of the F(ab’)2 fragment of Leo.H4 antibody at a dose of 20 μg per mouse just before the IVC ligation procedure.

### Quantification of NETs

Plasma NETs were measured using an ELISA-based assay. Briefly, an anti-MPO antibody (Santa Cruz Biotechnology, sc-390109) was coated on a 96-well plate followed by blocking the non-specific binding sites with 5% BSA. 15 μl of plasma and 35 μl of PBS were added into each well and incubated for 2 hours at room temperature under shaking conditions. After washing, the attached MPO-DNA complexes in the wells were quantified by an anti-DNA antibody conjugated with peroxidase using a commercial ELISA kit (Roche, Cat#11774425001).

To evaluate NET release *in vitro*, the endothelial cell monolayer was primed with TNF-α (100 ng/ml), to which neutrophils and platelets isolated from mice were added either separately or jointly with a ratio of 1:25, with or without the indicated agonist and antibody, and incubated for 2 hours at 37°C. After incubation, cells were treated with micrococcal nuclease to detach the released NETs from neutrophils, and NETs were further quantified using the Sytox green assay [25].

### Statistics

Data were analyzed using GraphPad Prism software program or Microsoft Excel. All results are presented as the mean ± SD. Statistical significance was calculated using a two-tailed Student’s *t*-test. More than two groups were compared using the One-way ANOVA post hoc test. A value of P < 0.05 was considered significant.

## Supplementary Material

Supplementary Figures
