# Supplementary material for: Kindlin-3 in platelets and myeloid cells differentially regulates deep vein thrombosis in mice
Source: Aging (Albany NY). 2019 Aug 31;11(17):6951–9. doi: 10.18632/aging.102229 (PMC6756892; doi:10.18632/aging.102229)
Supplement: Supplementary Figures [file aging-11-102229-s001.pdf]

SUPPLEMENTARY FIGURES

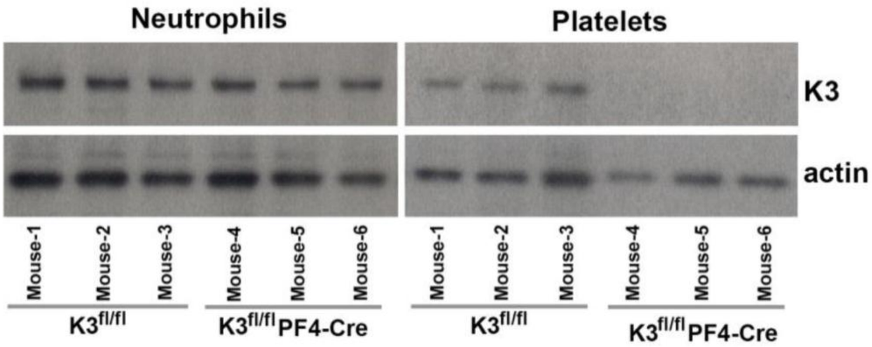

Supplementary Figure 1. The expression levels of kindlin-3 in neutrophils and platelets in Kindlin-3$^{fl/fl}$PF4-Cre mice. Bone marrow neutrophils and platelets were isolated from Kindlin-3$^{fl/fl}$PF4-Cre mice (K3$^{fl/fl}$PF4-Cre) and Kindlin-3$^{fl/fl}$ littermates (K3$^{fl/fl}$). The expression levels of kindlin-3 (K3) in these cells were evaluated by Western blotting using an anti-kindlin-3 antibody.

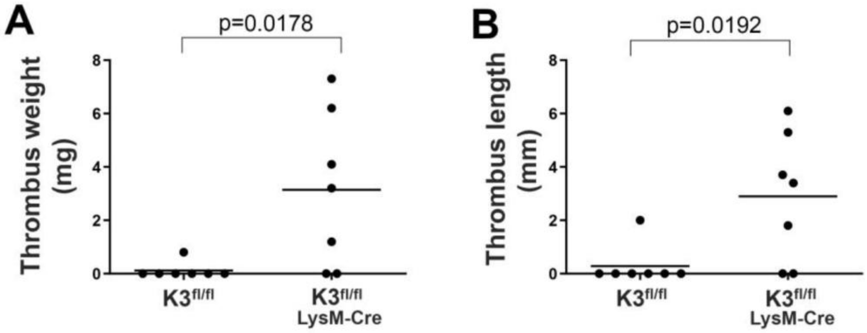

Supplementary Figure 2. Stenosis-induced DVT in Kindlin-3$^{fl/fl}$LysM-Cre mice. Kindlin-3$^{fl/fl}$LysM-Cre mice (K3$^{fl/fl}$LysM-Cre) and Kindlin-3$^{fl/fl}$ littermates (K3$^{fl/fl}$) were subjected to the partial IVC ligation for 2 hours. After that, the IVC tissues were harvested, and thrombus weight and length were evaluated; n = 7 for each group. A value of $P < 0.05$ was considered significant.
